# Supporting mental health during the COVID-19 pandemic: implementation of an e-guide

**DOI:** 10.1192/bjo.2021.517

**Published:** 2021-06-18

**Authors:** Athanasios Hassoulas, Eliana Panayiotou, Srinjay Mukhopadhyay, Ravanth Baskaran, Nan Zhang

**Affiliations:** 1 Cardiff University School of Medicine; 2 Swansea Bay University Health Board

## Abstract

**Aims:**

The COVID-19 pandemic has caused significant disruption to activities of daily living, which in turn has had a profound impact on the mental wellbeing of the public. An e-guide was designed to provide remote support to the general public through the application of a Behavioural Activation approach. Interactive, brief evidence-based exercises were included in the e-guide, along with mood ratings after each exercise to assess any improvements observed.

**Method:**

The e-guide was designed using the Xerte On- Line Toolkits open source software. Videos and interactive exercises were embedded within the resource, forming part of the brief intervention based on cognitivist and behaviourist principles. Information and further support was also provided for young people and parents. Videos from the public highlighting their experiences during the pandemic were also sourced and included (with consent). A pilot was launched to assess the impact of the e-guide. Participants were recruited from Cardiff University, mental health services and a local charity.

**Result:**

The e-guide was piloted on a sample of volunteers (n = 3), who completed a brief survey after engaging with the resource. Following the results of the pilot, the e-guide was promoted by the university's marketing team and made available to the public. At the 6-month mark, the e-guide had been accessed by 3228 individuals throughout the UK.

**Conclusion:**

The e-guide has since been disseminated by support services for young people, places of employment and eduction institutions. The national impact of the e-guide is evidenced from the number of people accessing the resource exceeding 3000. With the long-term effects of the pandemic taking hold, it remains crucial to support the wellbeing of the general public through such initiatives that are administered remotely

